# Potential of Bioactive Glasses for Cardiac and Pulmonary Tissue Engineering

**DOI:** 10.3390/ma10121429

**Published:** 2017-12-15

**Authors:** Saeid Kargozar, Sepideh Hamzehlou, Francesco Baino

**Affiliations:** 1 Department of Modern Sciences and Technologies, School of Medicine, Mashhad University of Medical Sciences, Mashhad 917794-8564, Iran; 2 Department of Medical Genetics, School of Medicine, Tehran University of Medical Sciences, Tehran 14155-6447, Iran; sepidy88@hotmail.com; 3 Institute of Materials Physics and Engineering, Department of Applied Science and Technology (DISAT), Politecnico di Torino, 10129 Torino, Italy

**Keywords:** bioactive glasses, scaffold, angiogenesis, soft tissue engineering, cardiac regeneration, lung tissue engineering

## Abstract

Repair and regeneration of disorders affecting cardiac and pulmonary tissues through tissue-engineering-based approaches is currently of particular interest. On this matter, different families of bioactive glasses (BGs) have recently been given much consideration with respect to treating refractory diseases of these tissues, such as myocardial infarction. The inherent properties of BGs, including their ability to bond to hard and soft tissues, to stimulate angiogenesis, and to elicit antimicrobial effects, along with their excellent biocompatibility, support these newly proposed strategies. Moreover, BGs can also act as a bioactive reinforcing phase to finely tune the mechanical properties of polymer-based constructs used to repair the damaged cardiac and pulmonary tissues. In the present study, we evaluated the potential of different forms of BGs, alone or in combination with other materials (e.g., polymers), in regards to repair and regenerate injured tissues of cardiac and pulmonary systems.

## 1. Introduction

From a medical point of view, disorders related to heart and pulmonary systems are considered as life-threatening pathologic conditions due to the high mortality rate [1,2]. This rate has been continuously growing as result of increased smoking and tobacco use, physical inactivity, and overweight and obesity [3]. Up to now, several approaches have been proposed and applied to treat the related disorders such as organ transplantation [4]. However, there are a couple of difficulties with regards to heart and lung transplantation, including antibody-mediated arterial lesions, antibody-mediated rejection, and infection [5,6,7]. With reference to these conditions, modern therapies have emerged in the effort to remove or at least partially overcome these complications. 

As advanced approaches, tissue engineering-based therapies have been considered for treating various disorders related to heart and pulmonary system [8,9,10]. To develop optimal tissue substitutes for the aforesaid tissues, different cells and biomaterials have been evaluated and used. From a cellular point of view, the use of adult stem cells has achieved a lot of attention [11,12]. As an illustration, it has been shown that transplantation of adult bone-marrow-derived cells to patients with myocardial infarction results in a statistically significant improvement in left ventricular ejection fraction (LVEF) [13]. From the perspective of the biomaterials, polymers are used more than other “rigid” materials (e.g., metals and ceramics) because of the soft texture of both heart and lung tissues [14,15,16]. However, these polymeric matrices need to be reinforced by other substances to accelerate repair and regeneration processes. For example, improving the transportation of nutrients and oxygen by adding angiogenesis-inducing materials to polymers is considered as one of the most promising strategies proposed for accelerated tissue reconstruction [17]. In the field of tissue engineering, various methods have been proposed and developed to stimulate angiogenesis; in this regard, one of the most recent and promising approaches involves the use of bioactive glasses (BGs) [18]. As previously mentioned in the literature, the strategies of inducing angiogenesis via biomaterials are feasible and effective and allow overcoming the limitations of “biological” approaches based on recombinant DNA technology, such as tumorigenicity and prolonged protein expression [19]. 

BGs have gained much attention in the field of tissue engineering and regenerative medicine over the last decades due to their highly attractive properties such as great biocompatibility and bioactivity [20]. These synthetic biomaterials were firstly discovered by Larry Hench and coauthors at the University of Florida in 1969 [21]. Although the first developed BG, the well-known 45S5 Bioglass^®^, was synthesized using a melt-quench route, later the sol-gel method was also used as an alternative approach to synthesize various types of new BG compositions [22]. Based on the glass network former, BGs can be categorized as silicate-based glasses, borosilicate and borate-based glasses, and phosphate-based glasses; basic compositions can be doped with metallic ions eliciting specific therapeutic effects once released in situ. Looking at the microstructure, “bulk” (i.e., non-porous) or inherently mesoporous glasses can be produced [23,24,25]. Nowadays, BGs are recognized as the second generation of biomaterials with the ability to bond to the living tissues [26]. Many in vitro and in vivo studies have been conducted on BGs to determine their potential for the reconstruction of different parts of human body, especially in orthopedic and dental applications [27,28]. Given the many satisfactory results, several types of BG-based products currently exist in the market, such as 45S5 Bioglass^®^, which are being used for hard tissue substitution to heal bony damages and defects in orthopedics and dentistry [29]. The main idea of using BGs for orthopedic applications comes from their ability to bond to bone tissue (high bioactivity) as well as osteoconduction and osteoinduction [30,31]. As previously mentioned in the literature [32,33], the release of various ions from the BGs structure into the biological environment influences cell response at the genetic level and, therefore, dictates their effects on the living cells and tissues (see Table 1). 

Although BGs have been used in the field of bone reconstruction, there are still some critical limitations for their widespread use, such as high brittleness and low compressive strength [51]. Therefore, a new trend has recently emerged among researchers to use these synthetic biomaterials for soft tissue regeneration [52]. In addition to inducing angiogenesis, there exist some additional reasons supporting the idea that BGs are suitable for soft tissues healing. For instance, it has been well documented that BGs are able to inhibit the growth of Gram-positive and Gram-negative bacteria [53,54]. This bactericidal activity is related to increasing local pH of the environment and generating reactive oxygen species (ROS) dictated by the release of ions (e.g., zinc) from the BG structure [55].

More recently, the use of BGs has been extended to other soft tissues including cardiac and pulmonary systems [56]. Similar to other soft tissues, the angiogenic effects of these relatively inexpensive materials are favorable for accelerating the healing procedures. From a general viewpoint, bonding of BGs to tissues is governed by dissolution and precipitation reactions at the implant surface consequent to contact with physiological fluids such as human plasma [21,57]. It is known that collagenous components of soft tissues are able to attach to BG surfaces, and it is also important to notice that progenitor cell populations in the surrounding tissues can partially affect the bonding and rate of regeneration [58]. 

**Figure 1:**
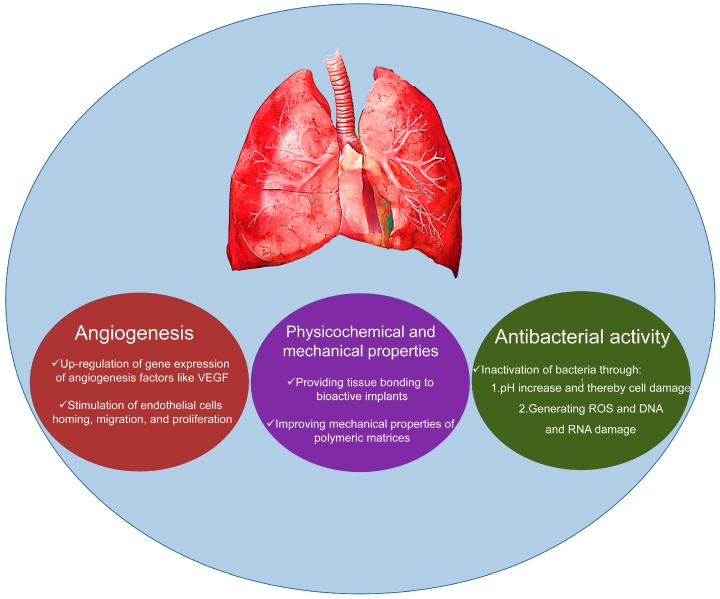
Schematic representation of potential of BGs for cardiac and pulmonary tissue engineering.

In this review, which is dedicated to the memory of Prof. Dame Julia Polak [59] (see the Acknowledgements), we aim to present the potential of BGs as effective materials to accelerate the regeneration of cardiac and pulmonary tissues also through stimulation of angiogenesis (Figure 1). For this purpose, we have collected and shortly discussed the existing literature about the BGs applied for regenerative strategies of the two aforementioned tissues, highlighting the critical issues and the challenges for future research. 

## 2. Applications in Cardiac Tissue Engineering

**Figure 2:**
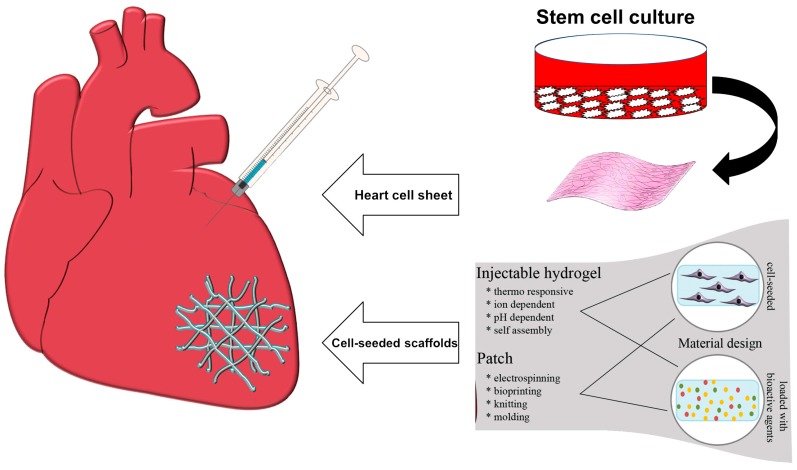
Examples of two approaches to cardiac tissue engineering. Stem cells are differentiated into cardiac repair cells and seeded onto a scaffold material or mixed with an injectable matrix. The scaffold with the repair cells is placed at the site of MI or used to replace the infarcted tissue. Cells encapsulated within a hydrogel matrix are delivered to the site of injury via direct injection. Images from Hinderer et al. [66] © Wiley Publishers.

Myocardial Infarction (MI), commonly known as a heart attack, is one of the most common cardiovascular disorders resulting in many deaths globally every year [60]. This disorder is caused by the blockage of the coronary arteries supplying the myocardium. The irreversible necrosis of heart muscle is the result of MI which needs to be repaired [61]. It has been previously identified that the human cardiomyocytes could not regenerate the damaged sites after MI [62]. Therefore, tissue engineering-based constructs such as injectable scaffolds (e.g., hydrogels) and engineered cardiac patches are being increasingly investigated and developed as effective tools to regenerate the damaged tissue [63,64]. As depicted in Figure 2, cardiac tissue engineering combines isolated functional cardiomyocytes and a biodegradable or non-degradable biomaterial to repair diseased heart muscle; a comprehensive overview on the issues and challenges associated to this research topic was provided by Chen et al. [65].

BGs were recently claimed as suitable materials for cardiac tissue engineering, especially in the form of nanoparticles in combination with biocompatible polymers acting as soft matrices (Table 2). The use of nano-sized BGs has recently increased and is now being extensively investigated in biomedicine [67,68,69]. The reasons are related to their high surface area and bioactivity, surface effects and biocompatible ion dissolution products that can also promote therapeutic effects in vitro and in vivo [70,71,72]. Moreover, nano-scale BGs can be easily dispersed in various polymeric systems, which make them proper materials to fabricate high-added-value composites for use in soft tissue engineering applications.

In the effort to prepare a heart patch, Chen et al. developed a series of elastomeric nanocomposites comprising a soft elastomer (poly(glycerol sebacate), PGS) and nanoparticles of 45S5 Bioglass^®^ (BG concentrations ranging from 0 to 10 wt %), from which alkaline cations were released upon soaking in aqueous media [73]. PGS has a range of drawbacks including limitations in mechanical properties, cytotoxicity caused by the acidic degradation products (at least in some types of PGS) and degradation kinetics that are too fast in vivo to provide sufficient mechanical support to the regenerated tissue. The glass was incorporated into the polymer to decrease the acidity resulted from the degradation of PGS without severely compromising its compliance. This could effectively improve the biocompatibility of the developed PGS–nanoBioglass^®^ (<5 wt %) composites. A mechanical improvement was also reported by the authors in the samples containing 45S5 Bioglass^®^ nanopowders that could allow the compliance of the constructs to satisfy the mechanical requirements of biomaterials applied in cardiac tissue engineering applications. This positive effect has been previously reported by other researchers that showed that the combination of nano-scale BGs with biopolymers is a good idea to improve their mechanical properties [77]. The authors reported the in vitro tests using fibroblasts and human endometrial stromal cell (ESC)-derived cardiomyocytes (hESC-CM), and their results confirmed that the PGS–nanoBioglass^®^ composites can enhance biocompatibility in comparison to the samples without glass particles. Moreover, beating rates for hESC-CM cultured in a medium containing PGS–nanoBioglass^®^ were similar to those of cells cultured in the standard culture medium at different time points, thus indicating that the nanocomposites do not inhibit the functional activity of the cells. The authors finally concluded that the nanocomposites are appropriate candidates for the development of palliative treatment of congestive heart failure through the cardiac patch strategy. 

The same research group also developed a set of PGS-based elastomeric composites containing micrometric 45S5 Bioglass^®^ fillers (5 μm-sized particles) [78]. In addition to much improved cytocompatibility due to the buffering effect of 45S5 Bioglass^®^ in preventing the rapid drop of pH through the release of alkaline ions (as already observed in [73]), these PGS-based composites demonstrated attractive mechanical properties including an increment of the elongation at break from 160% to 550% and an enhancement of the Young’s modulus from 0.38 to 1.62 MPa. It was also noted that the addition of 45S5 Bioglass^®^ in the composites mitigated the drop of the elastic modulus in a physiological environment with respect to PGS alone; interestingly, after the drop in the elastic modulus—which still occurred—the composites exhibited mechanical stability over an extended period. This last finding is a crucial feature of the new composites because they can provide reliable mechanical support to damaged soft tissues during the late stage of the healing process. 

In a very recent study by Taimoor et al. [79], novel electrically conductive polyaniline (PANI)/PGS composites were proposed as biocompatible scaffolds for cardiac tissue engineering and it was observed that the addition of PANI to PGS had a similar effect to that of 45S5 Bioglass^®^ in mitigating the drop in pH while material degradation occurs [78].

**Figure 3:**
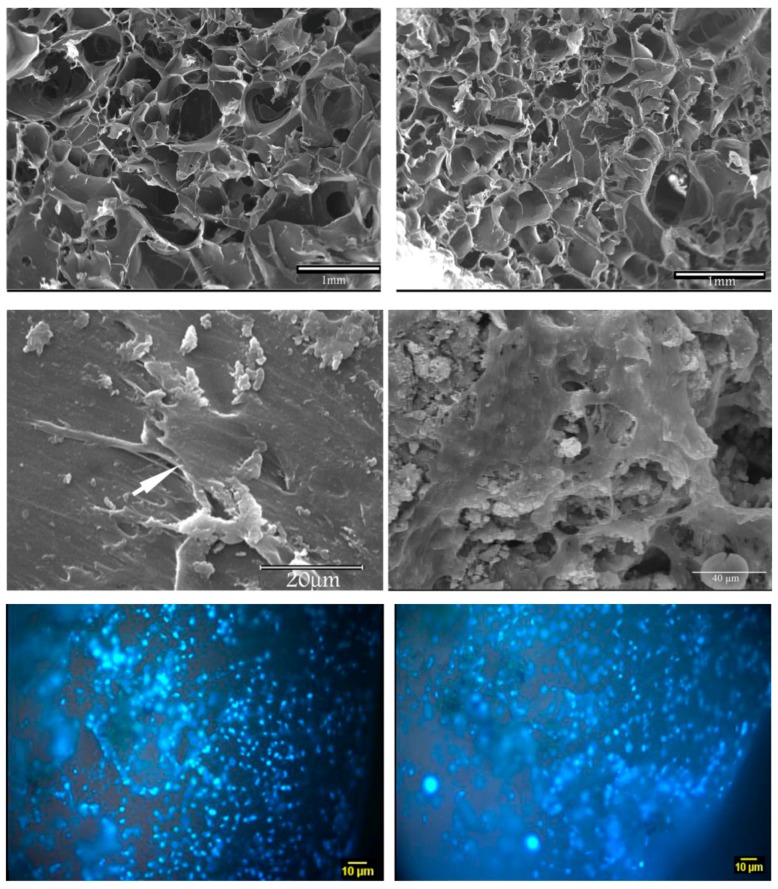
SEM micrographs of (**left**) gelatin/collagen scaffolds and (**right**) gelatin/collagen/BG scaffolds for cardiac tissue repair. Top: Pictures of bare scaffolds; middle: Cells seeded on the scaffolds; bottom: Nuclei staining of cells grown on scaffolds 15 days post cell seeding. Images from Barabadi et al. [74] © Elsevier.

As previously mentioned, the ionic dissolution products of BGs can stimulate various cell-like fibroblasts to secrete large amounts of angiogenic factors and cytokines resulting in the infiltration of vessels into the engineered scaffolds [80,81]. To evaluate this potential for cardiac tissue engineering strategies, Barabadi and co-workers fabricated hydrogel-based nanocomposite scaffolds containing BG nanoparticles for myocardial tissue engineering [74]. They explained that one of the main problems in myocardial tissue engineering is lack of functional vessels which ultimately results in a low survival rate of engineered tissues/injected cells [74]. Thus, they first synthesized sol-gel based BGs consisting of 45% SiO_2_, 24.5% CaO, 24.5% Na_2_O and 6% P_2_O_5_ (mol %) and then fabricated two types of hydrogel-based scaffolds including a gelatin-collagen hydrogel (Gel/Col) and a gelatin-collagen hydrogel containing BG nanoparticles (Gel/Col/BG). To show the effectiveness of the incorporated BGs on angiogenesis, they evaluated the differentiation capacity of undifferentiated human ESCs within the heterogeneous population of treated cells toward endothelial lineage (Figure 3). Their results indicated that the expression of vascular endothelial growth factor (VEGF) was significantly increased in the cells incubated with the conditioned media containing Gel/Col/BG scaffold compared to control. These data indicate that BG inclusion in hydrogel scaffold promotes the expression of VEGF and thereby enhances angiogenesis. The authors finally concluded that hydrogel scaffolds containing BGs are suitable constructs for myocardial tissue engineering since they are biocompatible and can promote angiogenesis.

**Figure 4:**
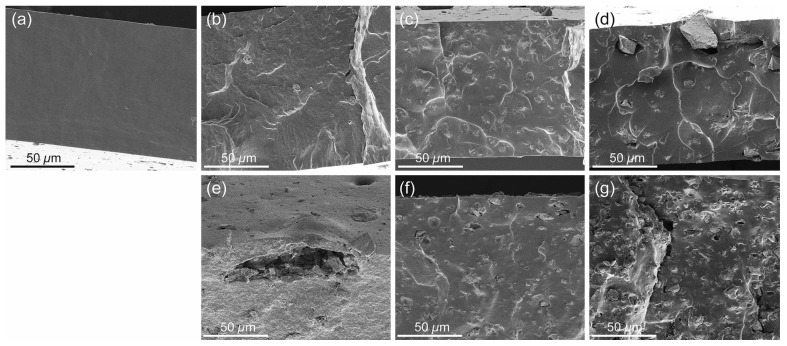
Cross-sectional SEM images of elastomeric biomaterials (silicones containing or not 10 wt % bioactive glass (BG45S5) particles) for cardiac tissue repair: (**a**) pure silicone; (**b**) silicone with nano-sized bioactive glass (nano-BG); (**c**) silicone with BG microparticles by Schott (Schott-BG); and (**d**) silicone with BG microparticles by Mo-Sci-Corporation (Mo-Sci-BG); and (**e**–**g**) the respective BG-containing composite films after four weeks of immersion in simulated body fluid (SBF). Images from Cohrs et al. [75] © Springer.

Cohrs and co-workers evaluated the role of incorporating of 45S5 BG nano- and micro-particles into silicone elastomers in order to improve the characteristics of left ventricular assist device (LVAD) drivelines [75]. The authors prepared three different nano- or micro-particles of 45S5 Bioglass^®^ by using flame spray synthesis and then incorporated them into medical-grade silicone elastomer to prepare thin composite films (Figure 4). They showed that incorporation of nano-BG particles into silicone composites led to the highest bioactivity and least swelling by 50% in the samples; however, these nano-particles caused lower mechanical properties as well as lower cytocompatibility. In contrast, micro-sized BG particles were twice as cytocompatible as nano-sized particles and possessed better mechanical properties and easier handling. However, the authors of this study concluded that the use of nano-sized glass particles in the case of driveline material for LVAD implantation was more advantageous than micro-sized particles due to higher bioactivity and less swelling inside the body. In vivo toxicity studies about the effect of BGs on the living heart are still rare, but preliminary results show promise [76].

## 3. Applications in Lung Tissue Engineering

Lung tissues have a poor self-regenerative ability and, thus, the only option for patients who suffer from severe chronic obstructive pulmonary disease (COPD)—often combined with pulmonary hypertension—is a transplant of heart and lungs [82]. 

The potential suitability of BGs for the repair of injured lung tissue (Table 3) was first investigated by a joint research group coordinated by Hench and Polak in the early 2000s: In this pioneering study, Tan et al. [83] functionalized the surface of sol-gel 58S glass scaffolds with amine or mercaptan groups and/or laminin and studied the in vitro biocompatibility of these glass-based foams with murine lung epithelial cells (MLE-12). The scope of the work was to find the best conditions to stimulate the growth and proliferation of lung cells, in the effort to pose the basis for developing tissue-engineered BG constructs for the repair of the pulmonary system. It was reported that lung cells colonized all types of porous scaffolds also in the core and, specifically, the laminin-coated and amine-modified glass foams were the most effective in promoting cell adhesion and growth. Sol-gel 58S glass was also found biocompatible towards human lung adenocarcinoma A549 cells [84].

**Figure 5:**
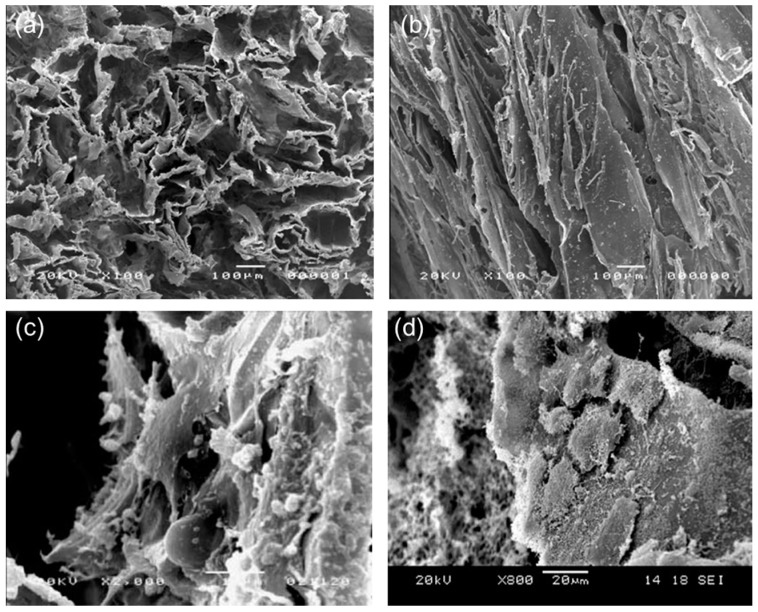
PDLLA/45S5 Bioglass^®^ scaffolds for possible application in lung tissue engineering: SEM micrographs showing the microstructure of the polymeric foam filled with 40 wt % of glass in sections: (**a**) orthogonal to pore direction; and (**b**) parallel to pore direction; and A549 cells seeded on the same scaffolds and incubated for: (**c**) three days; and (**d**) six days. Images from Verrier et al. [85] © Elsevier.

**Figure 6:**
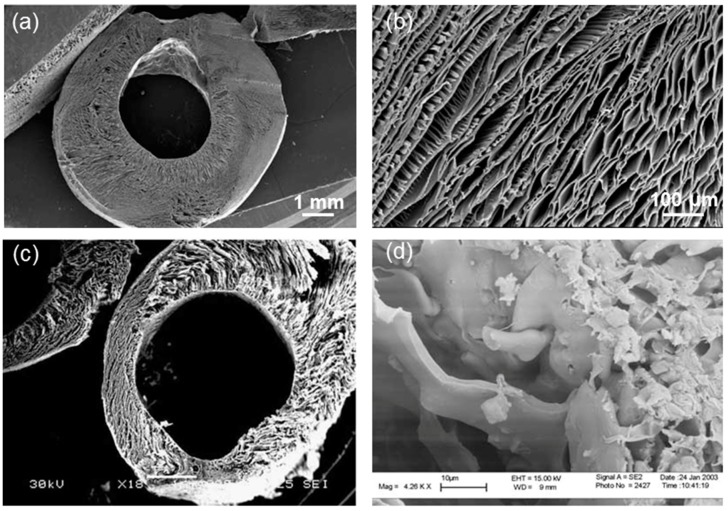
Tubular PLGA/45S5 Bioglass^®^ composite constructs: SEM micrographs showing: a radial section of an uncoated PLGA tube (**a**); and a detail of the cross-section (**b**); and the radial section of the composite tubular foam (**c**); and detail of the Bioglass^®^ particles on a fracture surface (**d**). Images from Boccaccini et al. [86] © Elsevier.

Polak and co-workers also explored the possibility of growing lung cells on porous poly(DL-lactic acid) (PDLLA) scaffolds. It was reported that PDLLA was not only non-toxic to pneumocytes but also actively supported the cell growth; these initial findings suggested the potential of this polymer to act as an appropriate matrix for engineering of distal lung tissue [89]. Following these seminal results, Verrier et al. [85] proposed the use of PDLLA/45S5 Bioglass^®^ composites for lung tissue engineering and performed in vitro biocompatibility assays with human lung carcinoma A549 cells (Figure 5); the idea behind this work was to take the best from the two materials (polymer and glass) that make up the porous composite. Two hours after cell seeding, a general increase of cell adhesion according to the increased content of 45S5 Bioglass^®^ (0 wt %, 5 wt % and 40 wt %) in the PDLLA foams was observed, but cell proliferation studies over a period of four weeks revealed a better aptitude of A549 cells to proliferate on scaffolds containing only 5 wt % of the glass. This result demonstrates that the concentration of BG in tissue engineering polymer-based constructs should be always optimized depending on the considered tissues that we want to regenerate. This dose-dependent effect was also observed in another study by the same research group, in which poly(lactic-*co*-glycolic acid) (PLGA)/45S5 Bioglass^®^ composite tubular foam scaffolds (porosity about 93 vol %, size of interconnected macropores in the 50–300 μm range, wall thickness within 1.5–3.0 mm) were fabricated via thermally induced phase separation [86]; the authors proposed the use of the produced constructs for the regeneration of tissues requiring a tubular shape scaffold (Figure 6), such as blood vessels and trachea.

It was also reported that doping of 58S sol-gel glass with moderate amounts of silver (2 mol % Ag_2_O max.) to obtain an antibacterial BG elicited no toxic effects towards A549 cells in vitro [87]. 

Wang et al. [88] demonstrated that the number of A549 cells can significantly decrease under the hyperthermic effect induced by Fe-doped sol-gel BGs due to the elevation of the temperature in the culture medium to 45 °C, which potentially opens new possibilities for treating lung carcinoma. 

The systemic toxicity of selected BGs and BG-containing composites was evaluated in some interesting studies carried out in vivo (rabbits). Taken together, chemical and histopathological analyses revealed neither morphological damage nor accumulation of ions (especially silicon) released from BGs in key organs such as brain, kidney, liver, spleen, heart, and lungs [76,90,91]. 

These set of results seems to actually support the fascinating possibility of using BG-based biomaterials and scaffolds in lung tissue engineering approaches, although extensive work, including testing with all the different cell types found in the pulmonary system, is necessary for further advancements.

## 4. Discussion and Future Challenges

This year, we are celebrating the 50th anniversary of the so-called “hypothesis of bioactive glass” [92]: In fact, before 1967, the concept of a material that could form a direct bond to living tissues seemed impossible. In 1985, Hench’s 45S5 Bioglass^®^ was approved by Food and Drug Administration (FDA) for clinical use in the repair of middle ear small bones and, since then, it has been successfully implanted in millions of patients worldwide to repair bone and dental defects [93]. The “second life” of BGs for potential applications in soft tissue regeneration is more recent [52,56] and, specifically, the first reported uses of these materials for lung and cardiac tissue engineering date back to 2003 [83] and 2010 [73], respectively. Indeed, more research still is to be carried out to support and expand the applications of BGs in these two highly-significant fields, albeit the available results show promise.

First, we need to understand and establish clearly what is the most appropriate way to use BGs in contact with cardiac and lung tissues. Looking at the BG-based products for bone repair that are currently available on the market, we can found BG particles, granules, moldable putties as well as 3D porous scaffolds that play a key role in tissue engineering approaches. 

The physico-mechanical characteristics of an ideal porous scaffold for bone tissue engineering have been established over the last 15 years taking cancellous bone as a reference tissue, and hence target ranges for pore and strut size, permeability, stiffness and compressive strength have been proposed in the literature [94,95,96]. Moving towards non-osseous applications, a crucial question is: What are the characteristics that a scaffold for cardiac or lung tissue engineering should exhibit? A response can be attempted, on the basis and within the frame of current knowledge. The specific physical properties that a cardiac tissue engineering construct is expected to have are: (i) biocompatibility; (ii) ability to promote cell adhesion and proliferation; (iii) tailored degradation rate matching the recovery rate of host soft tissue; (iv) permeability allowing the diffusion of biomolecules; (v) suitable mechanical properties close to those of natural tissues; (vi) contractility; and (vii) electrophysiological stability [97]. Properties (i)–(v) can be extended to lung tissue engineering constructs, too. Especially, Properties (v) and (vi) can be fulfilled only by using polymers, which means that BGs are suitable for these applications only if combined with a soft polymeric matrix. Besides being useful to tune the mechanical properties of the composite to match the features of the soft tissue to repair, BGs can play a major role from chemical and biological viewpoints, e.g., mitigation of pH drop due to polymer degradation and improved cytocompatibility. In this regard, there is a need for a significant amount of research to be carried out for establishing the specific mechanisms involved in the interaction between BGs and the cells that compose cardiac and lung tissues, just as performed for bone/dental applications in the last decades. At present, the role of BGs in promoting cardiac and lung cell proliferation and/or differentiation is as yet unknown. Basic cell and molecular biology studies on how BGs can actually stimulate cardiomyocytes and pneumocytes are still to be done, and this will require a close collaboration among glass chemists, biomaterials scientists, bioengineers, biologists, and clinicians. Future research will also require implementation of in vitro culture and co-culture strategies for the relevant cells found in these complex tissues that should be seeded on potentially-suitable BG-containing polymeric matrices. Suitable in vivo models will have to be defined and standardized, too. A careful selection of appropriate BG compositions and morphologies (e.g., micro- or nano-sized particles, and fibers) combined with suitable soft and porous polymeric matrices are necessary to realistically consider BGs in these particular fields.

The addition of metallic dopants to the BG composition, as well as the incorporation of growth factors and drugs in the composites, could be considered to develop new regeneration strategies for heart and lungs. An important limiting factor for progress in the field of regenerative medicine utilizing ions is the risk that they can easily diffuse to other non-target cells or tissues and, thus, stimulate unwanted responses. Mesoporous BGs (MBGs), which have been widely experimented as carriers for therapeutic ions and biomolecules and exhibit a controlled delivery capability for such agents [98], could partly overcome this problem by being embedded as high-added-value inclusions in biodegradable polymers. Many studies have been reported on the fabrication of MBG/polymer composites in the field of bone tissue engineering [99], but, to date, none of these constructs has been proposed for cardiac or lung tissue engineering. In general, applications in soft tissue engineering are dramatically scarce; only Jia et al. [100] suggested the use of MBG/chitosan composite films to induce hemostasis and accelerate wound healing.

Crucial questions need to be addressed before clinical translation, including: How can we minimize the nonspecific adverse effects of ions/drugs released by BG/MBG-based constructs? What is the underlying signaling cascade of these ions on cardiac/lung tissue regeneration? How can we combine advanced materials and stem cell sciences for new bone regenerative engineering?

Another aspect that deserves to be mentioned is the risk of soft tissue calcification: While the formation of a surface apatite layer (bioactivity) is key to generate a strong interfacial bond between BG and bone, this phenomenon should be avoided when BGs are put in contact with cardiac and lung tissues or, in general, soft organs of the body. 

Lastly, it will be crucial for developing constructs that can be easy-to-handle for surgeons and other clinical staff directly involved in their possible use and commercialization.

## 5. Conclusions

Taken together, BGs have achieved great interest in soft tissue engineering strategies over the recent years. The excellent biocompatibility, the ability to bond to living tissues, the antimicrobial properties and the ability to induce angiogenesis are considered as desirable characteristics of BGs to use in regenerative medicine [40]. Given these features, BGs are being currently used experimentally for soft tissue regeneration to find a way to use them clinically [101]. Up to now, most of these studies have been conducted on the skin and nerve tissues with satisfactory results [102,103,104,105]. Furthermore, the use of melt-derived and sol-gel BGs in the repair and regeneration of cardiac and lung tissues has also been reported with early promising results [73,74,75,76,83,84,85,86,87,88,89,90]. In this pioneering set of studies, BGs have been usually incorporated in polymeric matrices that match better the physico-mechanical properties of soft and delicate cardiac/lung tissues. Looking at the future, mesoporous BGs (MBGs) could be highly interesting platforms for the controlled release of therapeutic agents eliciting beneficial effects to injured heart and lungs [106]. MBGs could be indeed added to various biodegradable polymers to improve the functionality of the cardiac patches as well as to deliver desirable drugs and growth factors for accelerating regeneration. Overall, more research is needed to reveal all the aspects around the application of BGs in contact with these highly important tissues.

## Figures and Tables

**Table 1:** The main ions released from BGs considered for soft tissue engineering (adapted from Naseri et al. [34]).

Element	Effects	References
Si	-Promotes neovascularization. -Stimulates collagen type I formation.	[35,36]
Ca	-Promotes the migration and proliferation of epidermal cells. -Accelerates blood-clotting.	[37]
B	-Stimulates vascularization and angiogenesis. -Increases RNA synthesis in fibroblasts.	[38,39]
Cu	-Stimulates angiogenesis. -Antimicrobial property.	[40,41,42,43]
Ag^+^	-Anti-inflammatory property. -Antimicrobial property.	[44,45,46]
Zn	-Stimulates angiogenesis. -Enhances nerve regeneration. -Anti-inflammatory property. -Enhances wound healing processes.	[47,48,49]
Ga	-Antimicrobial property.	[50]

**Table 2:** Summary of the studies performed to evaluate the usability of BGs for heart tissue engineering.

Materials		In Vitro/In Vivo Tests	Remarks	References
BG Name and Composition	Polymeric Matrix			
45S5 Bioglass^®^ (45SiO_2_-24.5CaO-24.5Na_2_O-6P_2_O_5_ wt %) melt-derived particles	Poly(glycerol sebacate)	Human ESC derived cardiomyocytes	-Promoting cell differentiation	[73]
		Fibroblasts	-Non-toxic	
45S5 Bioglass^®^ (45SiO_2_-24.5CaO-24.5Na_2_O-6P_2_O_5_ wt %) melt-derived particles	Gelatin-collagen hydrogel	Endometrial stromal Stem cells (EnSCs)	-Inducing differentiation into cardiomyocytes -Increasing VEGF expression	[74]
		L929 cells	-Good biocompatibility	
45S5 Bioglass^®^ (45SiO_2_-24.5CaO-24.5Na_2_O-6P_2_O_5_ wt %) melt-derived particles	Silicone elastomer	Primary fibroblasts	-Improving mechanical properties -Improving cytocompatibility	[75]
Sol-gel BG nanoparticles (60SiO_2_-35CaO-5P_2_O_5_ mol %)	Gelatin/hyaluronic acid	Oral administration to rats	-No remarkable change in the morphology of heart tissue	[76]

**Table 3:** Summary of the studies involving the use of BGs for potential application in lung tissue engineering.

Materials		In Vitro/ In Vivo Tests	Remarks	References
BG Name and Composition	Polymeric Matrix			
58S (58SiO_2_-36CaO-6P_2_O_5_ mol %) sol-gel scaffold	-	MLE-12 cells	-Good biocompatibility further improved by laminin coating or amine functionalization	[83]
	-	A549 cells	-Good biocompatibility	[84]
45S5 Bioglass^®^ (45SiO_2_-24.5CaO-24.5Na_2_O-6P_2_O_5_ wt %) melt-derived particles	PDLLA	A549 cells	-Dose-dependent effect	[85]
	PLGA	L929 cells	-Dose-dependent effect	[86]
Ag-doped 58S sol-gel glass (58SiO_2_-(36-x)CaO-6P_2_O_5_-xAg_2_O, with x = 0, 1, 2 mol %)	-	A549 cells	-Non-toxic	[87]
Fe-doped sol-gel glass (basic composition: 8.4Na_2_O-40CaO-39.6SiO_2_-12P_2_O_5_ wt % doped with 0.2-1 wt % of Fe)	-	A549 cells	-Hyperthermic effect for possible application in lung cancer treatment	[88]
Sol-gel BG nanoparticles (60SiO_2_-35CaO-5P_2_O_5_ mol %)	Gelatin/hyaluronic acid	Oral administration to rats	-No remarkable change in the morphology of lung tissue	[76]

## References

[1] Dawber T.R., Moore F.E., Mann G.V. (2015). Coronary Heart Disease in the Framingham Study. Int. J. Epidemiol..

[2] Galiè N., Humbert M., Vachiery J.-L., Gibbs S., Lang I., Torbicki A., Simonneau G., Peacock A., Vonk Noordegraaf A., Beghetti M. (2015). 2015 ESC/ERS guidelines for the diagnosis and treatment of pulmonary hypertension: The joint task force for the diagnosis and treatment of pulmonary hypertension of the European Society of Cardiology (ESC) and the European Respiratory Society (ERS): Endorsed by: Association for European Paediatric and Congenital Cardiology (AEPC), International Society for Heart and Lung Transplantation (ISHLT). Eur. Heart J..

[3] Members W.G., Go A.S., Mozaffarian D., Roger V.L., Benjamin E.J., Berry J.D., Blaha M.J., Dai S., Ford E.S., Fox C.S. (2014). Heart disease and stroke statistics—2014 update: A report from the American Heart Association. Circulation.

[4] Hashimoto K., Miyoshi K., Mizutani H., Otani S., Sugimoto S., Yamane M., Oto T. (2017). Successful Lung Transplantation for Pulmonary Disease Associated With Erdheim-Chester Disease. Ann. Thorac. Surg..

[5] Levine D.J., Glanville A.R., Aboyoun C., Belperio J., Benden C., Berry G.J., Hachem R., Hayes D., Neil D., Reinsmoen N.L. (2016). Antibody-mediated rejection of the lung: A consensus report of the International Society for Heart and Lung Transplantation. J. Heart Lung Transplant..

[6] Loupy A., Toquet C., Rouvier P., Beuscart T., Bories M., Varnous S., Guillemain R., Pattier S., Suberbielle C., Leprince P. (2016). Late failing heart allografts: Pathology of cardiac allograft vasculopathy and association with antibody-mediated rejection. Am. J. Transplant..

[7] De Vlaminck I., Martin L., Kertesz M., Patel K., Kowarsky M., Strehl C., Cohen G., Luikart H., Neff N.F., Okamoto J. (2015). Noninvasive monitoring of infection and rejection after lung transplantation. Proc. Natl. Acad. Sci. USA.

[8] Scarritt M.E., Pashos N.C., Bunnell B.A. (2015). A review of cellularization strategies for tissue engineering of whole organs. Front. Bioeng. Biotechnol..

[9] Tseng H., Puperi D.S., Kim E.J., Ayoub S., Shah J.V., Cuchiara M.L., West J.L., Grande-Allen K.J. (2014). Anisotropic poly (ethylene glycol)/polycaprolactone hydrogel-fiber composites for heart valve tissue engineering. Tissue Eng. Part A.

[10] Nikolić M.Z., Johnson J.-A., Sun D., Caritg O., Laresgoiti U., Brady J., Allen G., Giangreco A., Rawlins E.L. (2017). Development of a genetically modifiable epithelial in-vitro culture system from human embryonic lung epithelial stem cells: Towards human lung regeneration in end-stage respiratory failure. Lancet.

[11] Garbern J.C., Lee R.T. (2013). Cardiac stem cell therapy and the promise of heart regeneration. Cell Stem Cell.

[12] Kotton D.N., Morrisey E.E. (2014). Lung regeneration: Mechanisms, applications and emerging stem cell populations. Nat. Med..

[13] Clifford D.M., Fisher S.A., Brunskill S.J., Doree C., Mathur A., Watt S., Martin-Rendon E. (2012). Stem cell treatment for acute myocardial infarction. Cochrane Database Syst. Rev..

[14] Camci-Unal G., Annabi N., Dokmeci M.R., Liao R., Khademhosseini A. (2014). Hydrogels for cardiac tissue engineering. NPG Asia Mater..

[15] Boffito M., Sartori S., Ciardelli G. (2014). Polymeric scaffolds for cardiac tissue engineering: Requirements and fabrication technologies. Polym. Int..

[16] Mahoney C., Conklin D., Waterman J., Sankar J., Bhattarai N. (2016). Electrospun nanofibers of poly (ε-caprolactone)/depolymerized chitosan for respiratory tissue engineering applications. J. Biomater. Sci. Polym. Ed..

[17] Leite Á.J., Mano J. (2017). Biomedical applications of natural-based polymers combined with bioactive glass nanoparticles. J. Mater. Chem. B.

[18] Kargozar S., Lotfibakhshaiesh N., Ai J., Mozafari M., Milan P.B., Hamzehlou S., Barati M., Baino F., Hill R.G., Joghataei M.T. (2017). Strontium- and cobalt-substituted bioactive glasses seeded with human umbilical cord perivascular cells to promote bone regeneration via enhanced osteogenic and angiogenic activities. Acta Biomater..

[19] Kargozar S., Hashemian S.J., Soleimani M., Milan P.B., Askari M., Khalaj V., Samadikuchaksaraie A., Hamzehlou S., Katebi A.R., Latifi N. (2017). Acceleration of bone regeneration in bioactive glass/gelatin composite scaffolds seeded with bone marrow-derived mesenchymal stem cells over-expressing bone morphogenetic protein-7. Mater. Sci. Eng. C.

[20] Kargozar S., Mozafari M., Hashemian S.J., Milan P.B., Hamzehlou S., Soleimani M., Joghataei M.T., Gholipourmalekabadi M., Korourian A., Mousavizadeh K. (2018). Osteogenic potential of stem cells-seeded bioactive nanocomposite scaffolds: A comparative study between human mesenchymal stem cells derived from bone, umbilical cord Wharton’s jelly, and adipose tissue. J. Biomed. Mater. Res. Part B Appl. Biomater..

[21] Hench L.L., Splinter R.J., Allen W., Greenlee T. (1971). Bonding mechanisms at the interface of ceramic prosthetic materials. J. Biomed. Mater. Res. Part A.

[22] Baghbani F., Moztarzadeh F., Hajibaki L., Mozafari M. (2013). Synthesis, characterization and evaluation of bioactivity and antibacterial activity of quinary glass system (SiO_2_-CaO-P_2_O_5_-MgO-ZnO): In vitro study. Bull. Mater. Sci..

[23] Kargozar S., Lotfibakhshaiesh N., Ai J., Samadikuchaksaraie A., Hill R.G., Shah P.A., Milan P.B., Mozafari M., Fathi M., Joghataei M.T. (2016). Synthesis, physico-chemical and biological characterization of strontium and cobalt substituted bioactive glasses for bone tissue engineering. J. Non-Cryst. Solids.

[24] Baino F., Fiorilli S.L., Mortera R.S., Onida B., Saino E., Visai L., Verné E., Vitale-Brovarone C. (2012). Mesoporous bioactive glass as a multifunctional system for bone regeneration and controlled drug release. J. Appl. Biomater. Funct. Mater..

[25] Baino F., Vitale-Brovarone C. (2015). Feasibility of glass-ceramic coatings on alumina prosthetic implants by airbrush spraying method. Ceram. Int..

[26] Vergnol G., Ginsac N., Rivory P., Meille S., Chenal J.M., Balvay S., Chevalier J., Hartmann D.J. (2016). In vitro and in vivo evaluation of a polylactic acid-bioactive glass composite for bone fixation devices. J. Biomed. Mater. Res. Part B Appl. Biomater..

[27] Johari B., Kadivar M., Lak S., Gholipourmalekabadi M., Urbanska A.M., Mozafari M., Ahmadzadehzarajabad M., Azarnezhad A., Afshari S., Zargan J. (2016). Osteoblast-seeded bioglass/gelatin nanocomposite: A promising bone substitute in critical-size calvarial defect repair in rat. Int. J. Artif. Organs.

[28] Johari B., Ahmadzadehzarajabad M., Azami M., Kazemi M., Soleimani M., Kargozar S., Hajighasemlou S., Farajollahi M.M., Samadikuchaksaraei A. (2016). Repair of rat critical size calvarial defect using osteoblast-like and umbilical vein endothelial cells seeded in gelatin/hydroxyapatite scaffolds. J. Biomed. Mater. Res. Part A.

[29] Hench L.L. (2013). Chronology of bioactive glass development and clinical applications. New J. Glass Ceram..

[30] Westhauser F., Weis C., Prokscha M., Bittrich L.A., Li W., Xiao K., Kneser U., Kauczor H.-U., Schmidmaier G., Boccaccini A.R. (2016). Three-dimensional polymer coated 45S5-type bioactive glass scaffolds seeded with human mesenchymal stem cells show bone formation in vivo. J. Mater. Sci. Mater. Med..

[31] Bellucci D., Anesi A., Salvatori R., Chiarini L., Cannillo V. (2017). A comparative in vivo evaluation of bioactive glasses and bioactive glass-based composites for bone tissue repair. Mater. Sci. Eng. C.

[32] Durand L.A.H., Vargas G.E., Romero N.M., Vera-Mesones R., Porto-López J.M., Boccaccini A.R., Zago M.P., Baldi A., Gorustovich A. (2015). Angiogenic effects of ionic dissolution products released from a boron-doped 45S5 bioactive glass. J. Mater. Chem. B.

[33] Guan J., Zhang J., Guo S., Zhu H., Zhu Z., Li H., Wang Y., Zhang C., Chang J. (2015). Human urine-derived stem cells can be induced into osteogenic lineage by silicate bioceramics via activation of the Wnt/β-catenin signaling pathway. Biomaterials.

[34] Naseri S., Lepry W.C., Nazhat S.N. (2017). Bioactive glasses in wound healing: Hope or hype?. J. Mater. Chem. B.

[35] Gerhardt L.C., Widdows K.L., Erol M.M., Nandakumar A., Roqan I.S., Ansari T., Boccaccini A.R. (2013). Neocellularization and neovascularization of nanosized bioactive glass-coated decellularized trabecular bone scaffolds. J. Biomed. Mater. Res. Part A.

[36] Reffitt D., Ogston N., Jugdaohsingh R., Cheung H., Evans B.A.J., Thompson R., Powell J., Hampson G. (2003). Orthosilicic acid stimulates collagen type 1 synthesis and osteoblastic differentiation in human osteoblast-like cells in vitro. Bone.

[37] Lansdown A.B. (2002). Calcium: A potential central regulator in wound healing in the skin. Wound Repair Regen..

[38] Durand L.A.H., Góngora A., López J.M.P., Boccaccini A.R., Zago M.P., Baldi A., Gorustovich A. (2014). In vitro endothelial cell response to ionic dissolution products from boron-doped bioactive glass in the SiO_2_-CaO-P_2_O_5_-Na_2_O system. J. Mater. Chem. B.

[39] Dzondo-Gadet M., Mayap-Nzietchueng R., Hess K., Nabet P., Belleville F., Dousset B. (2002). Action of boron at the molecular level. Biol. Trace Elem. Res..

[40] Li J., Zhai D., Lv F., Yu Q., Ma H., Yin J., Yi Z., Liu M., Chang J., Wu C. (2016). Preparation of copper-containing bioactive glass/eggshell membrane nanocomposites for improving angiogenesis, antibacterial activity and wound healing. Acta Biomater..

[41] Goh Y.F., Alshemary A.Z., Akram M., Kadir A., Rafiq M., Hussain R. (2014). Bioactive Glass: An In-Vitro Comparative Study of Doping with Nanoscale Copper and Silver Particles. Int. J. Appl. Glass Sci..

[42] Gérard C., Bordeleau L.-J., Barralet J., Doillon C.J. (2010). The stimulation of angiogenesis and collagen deposition by copper. Biomaterials.

[43] Rodríguez J.P., Rios S., Gonzalez M. (2002). Modulation of the proliferation and differentiation of human mesenchymal stem cells by copper. J. Cell. Biochem..

[44] Rai M., Yadav A., Gade A. (2009). Silver nanoparticles as a new generation of antimicrobials. Biotechnol. Adv..

[45] Nadworny P.L., Wang J., Tredget E.E., Burrell R.E. (2008). Anti-inflammatory activity of nanocrystalline silver in a porcine contact dermatitis model. Nanomed. Nanotechnol. Biol. Med..

[46] Chaloupka K., Malam Y., Seifalian A.M. (2010). Nanosilver as a new generation of nanoproduct in biomedical applications. Trends Biotechnol..

[47] Augustine R., Dominic E.A., Reju I., Kaimal B., Kalarikkal N., Thomas S. (2014). Investigation of angiogenesis and its mechanism using zinc oxide nanoparticle-loaded electrospun tissue engineering scaffolds. RSC Adv..

[48] Yin Y., Cui Q., Li Y., Irwin N., Fischer D., Harvey A.R., Benowitz L.I. (2003). Macrophage-derived factors stimulate optic nerve regeneration. J. Neurosci..

[49] Lang C., Murgia C., Leong M., Tan L.-W., Perozzi G., Knight D., Ruffin R., Zalewski P. (2007). Anti-inflammatory effects of zinc and alterations in zinc transporter mRNA in mouse models of allergic inflammation. Am. J. Physiol.-Lung Cell. Mol. Physiol..

[50] Valappil S.P., Ready D., Neel E.A.A., Pickup D.M., Chrzanowski W., O’Dell L.A., Newport R.J., Smith M.E., Wilson M., Knowles J.C. (2008). Antimicrobial gallium-doped phosphate-based glasses. Adv. Funct. Mater..

[51] Baino F., Ferraris M., Bretcanu O., Verné E., Vitale-Brovarone C. (2013). Optimization of composition, structure and mechanical strength of bioactive 3-D glass-ceramic scaffolds for bone substitution. J. Biomater. Appl..

[52] Baino F., Novajra G., Miguez-Pacheco V., Boccaccini A.R., Vitale-Brovarone C. (2016). Bioactive glasses: Special applications outside the skeletal system. J. Non-Cryst. Solids.

[53] Coraça-Huber D.C., Fille M., Hausdorfer J., Putzer D., Nogler M. (2014). Efficacy of antibacterial bioactive glass S53P4 against *S. aureus* biofilms grown on titanium discs in vitro. J. Orthop. Res..

[54] Stoor P., Frantzen J. (2017). Influence of bioactive glass S53P4 granules and putty on osteomyelitis associated bacteria in vitro. Biomed. Glasses.

[55] Zhang D., Leppäranta O., Munukka E., Ylänen H., Viljanen M.K., Eerola E., Hupa M., Hupa L. (2010). Antibacterial effects and dissolution behavior of six bioactive glasses. J. Biomed. Mater. Res. Part A.

[56] Miguez-Pacheco V., Hench L.L., Boccaccini A.R. (2015). Bioactive glasses beyond bone and teeth: Emerging applications in contact with soft tissues. Acta Biomater..

[57] Hench L.L. (2015). The future of bioactive ceramics. J. Mater. Sci. Mater. Med..

[58] Hench L.L., Greenspan D. (2013). Interactions between bioactive glass and collagen: A review and new perspectives. J. Aust. Ceram. Soc..

[59] Laurance J. (2014). Julia Margaret Polak. Lancet.

[60] Gerber Y., Weston S.A., Enriquez-Sarano M., Berardi C., Chamberlain A.M., Manemann S.M., Jiang R., Dunlay S.M., Roger V.L. (2016). Mortality associated with heart failure after myocardial infarction: A contemporary community perspective. Circ. Heart Fail..

[61] Hausenloy D.J., Yellon D.M. (2013). Myocardial ischemia-reperfusion injury: A neglected therapeutic target. J. Clin. Investig..

[62] Kikuchi K., Poss K.D. (2012). Cardiac regenerative capacity and mechanisms. Annu. Rev. Cell Dev. Biol..

[63] Rane A.A., Christman K.L. (2011). Biomaterials for the treatment of myocardial infarction: A 5-year update. J. Am. Coll. Cardiol..

[64] Reis L.A., Chiu L.L., Feric N., Fu L., Radisic M. (2016). Biomaterials in myocardial tissue engineering. J. Tissue Eng. Regen. Med..

[65] Chen Q.-Z., Harding S.E., Ali N.N., Lyon A.R., Boccaccini A.R. (2008). Biomaterials in cardiac tissue engineering: Ten years of research survey. Mater. Sci. Eng. R Rep..

[66] Hinderer S., Brauchle E., Schenke-Layland K. (2015). Generation and assessment of functional biomaterial scaffolds for applications in cardiovascular tissue engineering and regenerative medicine. Adv. Healthc. Mater..

[67] Vargas G.E., Durand L.A.H., Cadena V., Romero M., Mesones R.V., Mačković M., Spallek S., Spiecker E., Boccaccini A.R., Gorustovich A.A. (2013). Effect of nano-sized bioactive glass particles on the angiogenic properties of collagen based composites. J. Mater. Sci. Mater. Med..

[68] Hu Q., Li Y., Miao G., Zhao N., Chen X. (2014). Size control and biological properties of monodispersed mesoporous bioactive glass sub-micron spheres. RSC Adv..

[69] Lukowiak A., Lao J., Lacroix J., Nedelec J.-M. (2013). Bioactive glass nanoparticles obtained through sol-gel chemistry. Chem. Commun..

[70] Erol-Taygun M., Zheng K., Boccaccini A.R. (2013). Nanoscale bioactive glasses in medical applications. Int. J. Appl. Glass Sci..

[71] Ji L., Qiao W., Huang K., Zhang Y., Wu H., Miao S., Liu H., Dong Y., Zhu A., Qiu D. (2017). Synthesis of nanosized 58S bioactive glass particles by a three-dimensional ordered macroporous carbon template. Mater. Sci. Eng. C.

[72] Covarrubias C., Arroyo F., Balanda C., Neira M., von Marttens A., Caviedes P., Rodríguez J.P., Urra C. (2015). The effect of the nanoscale structure of nanobioceramics on their in vitro bioactivity and cell differentiation properties. J. Nanomater..

[73] Chen Q., Jin L., Cook W.D., Mohn D., Lagerqvist E.L., Elliott D.A., Haynes J.M., Boyd N., Stark W.J., Pouton C.W. (2010). Elastomeric nanocomposites as cell delivery vehicles and cardiac support devices. Soft Matter.

[74] Barabadi Z., Azami M., Sharifi E., Karimi R., Lotfibakhshaiesh N., Roozafzoon R., Joghataei M.T., Ai J. (2016). Fabrication of hydrogel based nanocomposite scaffold containing bioactive glass nanoparticles for myocardial tissue engineering. Mater. Sci. Eng. C.

[75] Cohrs N.H., Schulz-Schönhagen K., Jenny F., Mohn D., Stark W.J. (2017). Bioactive glass containing silicone composites for left ventricular assist device drivelines: Role of bioglass 45s5^®^ particle size on mechanical properties and cytocompatibility. J. Mater. Sci..

[76] Zhou Z., Xiang L., Ou B., Huang T., Zhou H., Zeng W., Liu L., Liu Q., Zhao Y., He S. (2014). Biological assessment in-vivo of gel-HA scaffold materials containing nano-bioactive glass for tissue engineering. J. Macromol. Sci. Part A.

[77] Boccaccini A.R., Erol M., Stark W.J., Mohn D., Hong Z., Mano J.F. (2010). Polymer/bioactive glass nanocomposites for biomedical applications: A review. Compos. Sci. Technol..

[78] Liang S.-L., Cook W.D., Thouas G.A., Chen Q.-Z. (2010). The mechanical characteristics and in vitro biocompatibility of poly (glycerol sebacate)-bioglass^®^ elastomeric composites. Biomater..

[79] Qazi T.H., Rai R., Dippold D., Roether J.E., Schubert D.W., Rosellini E., Barbani N., Boccaccini A.R. (2014). Development and characterization of novel electrically conductive pani-pgs composites for cardiac tissue engineering applications. Acta Biomater..

[80] Gorustovich A.A., Roether J.A., Boccaccini A.R. (2009). Effect of bioactive glasses on angiogenesis: A review of in vitro and in vivo evidences. Tissue Eng. Part B Rev..

[81] Detsch R., Stoor P., Grünewald A., Roether J.A., Lindfors N.C., Boccaccini A.R. (2014). Increase in VEGF secretion from human fibroblast cells by bioactive glass S53P4 to stimulate angiogenesis in bone. J. Biomed. Mater. Res. Part A.

[82] Petersen T.H., Calle E.A., Zhao L., Lee E.J., Gui L., Raredon M.B., Gavrilov K., Yi T., Zhuang Z.W., Breuer C. (2010). Tissue-engineered lungs for in vivo implantation. Science.

[83] Tan A., Romanska H., Lenza R., Jones J.R., Hench L.L., Polak J.M., Bishop A. (2003). The effect of 58S bioactive sol-gel derived foams on the growth of murine lung epithelial cells. Key Eng. Mater..

[84] Saravanapavan P., Verrier S., Hench L.L. (2004). A549 lung carcinoma cells: Binary vs. ternary bioactive gel-glasses. Key Eng. Mater..

[85] Verrier S., Blaker J.J., Maquet V., Hench L.L., Boccaccini A.R. (2004). PDLLA/Bioglass^®^ composites for soft-tissue and hard-tissue engineering: An in vitro cell biology assessment. Biomaterials.

[86] Boccaccini A.R., Blaker J.J., Maquet V., Day R., Jérôme R. (2005). Preparation and characterisation of poly (lactide-co-glycolide) (PLGA) and PLGA/Bioglass^®^ composite tubular foam scaffolds for tissue engineering applications. Mater. Sci. Eng. C.

[87] Pires E.G., Bonan R.F., Rocha Í.M., Gonçalves I.M.F., de Souza J.R., Gonzales L.H.V., Junior J.V.J.D., Perez D.E.D., Tavares P.C.B., da Silva S.M. (2017). Silver-doped 58S Bioactive Glass as an Anti-Leishmania Agent. Int. J. Appl. Glass Sci..

[88] Wang T.W., Wu H.C., Wang W.R., Lin F.H., Lou P.J., Shieh M.J., Young T.H. (2007). The development of magnetic degradable DP-Bioglass for hyperthermia cancer therapy. J. Biomed. Mater. Res. Part A.

[89] Lin Y., Boccaccini A., Polak J., Bishop A., Maquet V. (2006). Biocompatibility of poly-dl-lactic acid (pdlla) for lung tissue engineering. J. Biomater. Appl..

[90] Meseguer-Olmo L., Ros-Nicolás M., Clavel-Sainz M., Vicente-Ortega V., Alcaraz-Baños M., Lax-Pérez A., Arcos D., Ragel C., Vallet-Regí M. (2002). Biocompatibility and in vivo gentamicin release from bioactive sol-gel glass implants. J. Biomed. Mater. Res. Part A.

[91] Lai W., Garino J., Flaitz C., Ducheyne P. (2005). Excretion of resorption products from bioactive glass implanted in rabbit muscle. J. Biomed. Mater. Res. Part A.

[92] Hench L.L. (2006). The story of Bioglass^®^. J. Mater. Sci. Mater. Med..

[93] Jones J.R., Brauer D.S., Hupa L., Greenspan D.C. (2016). Bioglass and bioactive glasses and their impact on healthcare. Int. J. Appl. Glass Sci..

[94] Jones J.R., Gentleman E., Polak J. (2007). Bioactive glass scaffolds for bone regeneration. Elements.

[95] Gerhardt L.-C., Boccaccini A.R. (2010). Bioactive glass and glass-ceramic scaffolds for bone tissue engineering. Materials.

[96] Baino F., Vitale-Brovarone C. (2011). Three-dimensional glass-derived scaffolds for bone tissue engineering: Current trends and forecasts for the future. J. Biomed. Mater. Res. Part A.

[97] Tallawi M., Rosellini E., Barbani N., Cascone M.G., Rai R., Saint-Pierre G., Boccaccini A.R. (2015). Strategies for the chemical and biological functionalization of scaffolds for cardiac tissue engineering: A review. J. R. Soc. Interface.

[98] Wu C., Chang J. (2014). Multifunctional mesoporous bioactive glasses for effective delivery of therapeutic ions and drug/growth factors. J. Controll. Release.

[99] Baino F., Fiorilli S., Vitale-Brovarone C. (2017). Composite biomaterials based on sol-gel mesoporous silicate glasses: A review. Bioengineering.

[100] Jia T.-B., Chen J.-Y., Feng X.-X., Chang J. (2011). Symbolfabrication and characterization of chitosan/mesoporous bioactive glasses porous films. J. Clin. Rehabil. Tissue Eng. Res..

[101] Miguez-Pacheco V., Greenspan D., Hench L., Boccaccini A. (2015). Bioactive glasses in soft tissue repair. Am. Ceram. Soc. Bull..

[102] Lin C., Mao C., Zhang J., Li Y., Chen X. (2012). Healing effect of bioactive glass ointment on full-thickness skin wounds. Biomed. Mater..

[103] Zhao S., Li L., Wang H., Zhang Y., Cheng X., Zhou N., Rahaman M.N., Liu Z., Huang W., Zhang C. (2015). Wound dressings composed of copper-doped borate bioactive glass microfibers stimulate angiogenesis and heal full-thickness skin defects in a rodent model. Biomaterials.

[104] Souza M.T., Peitl O., Zanotto E.D., Boccaccini A.R. (2016). Novel Double-Layered Conduit Containing Highly Bioactive Glass Fibers for Potential Nerve Guide Application. Int. J. Appl. Glass Sci..

[105] Koudehi M.F., Fooladi A.A.I., Mansoori K., Jamalpoor Z., Amiri A., Nourani M.R. (2014). Preparation and evaluation of novel nano-bioglass/gelatin conduit for peripheral nerve regeneration. J. Mater. Sci. Mater. Med..

[106] Wu C., Fan W., Chang J., Xiao Y. (2013). Mesoporous bioactive glass scaffolds for efficient delivery of vascular endothelial growth factor. J. Biomater. Appl..

